# Differences in interaction lead to the formation of different types of insulin amyloid

**DOI:** 10.1038/s41598-022-12212-6

**Published:** 2022-05-20

**Authors:** Wakako Mori, Ryosuke Kawakami, Yosuke Niko, Tomohiro Haruta, Takeshi Imamura, Kentaro Shiraki, Tamotsu Zako

**Affiliations:** 1 Department of Chemistry and Biology, Graduate School of Science and Engineering, Ehime University, Ehime, Japan; 2 Department of Molecular Medicine for Pathogenesis, Graduate School of Medicine, Ehime University, Ehime, Japan; 3 Research and Education Faculty, Multidisciplinary Science Cluster, Interdisciplinary Science Unit, Kochi University, Kochi, Japan; 4 Application Management Department, JEOL Ltd, Tokyo, Japan; 5 Faculty of Pure and Applied Sciences, University of Tsukuba, Ibaraki, Japan

**Keywords:** Protein aggregation, Peptides

## Abstract

Insulin balls, localized insulin amyloids formed at the site of repeated insulin injections in patients with diabetes, cause poor glycemic control and cytotoxicity. Our previous study has shown that insulin forms two types of amyloids; toxic amyloid formed from the intact insulin ((i)-amyloid) and less-toxic amyloid formed in the presence of the reducing reagent TCEP ((r)-amyloid), suggesting insulin amyloid polymorphism. However, the differences in the formation mechanism and cytotoxicity expression are still unclear. Herein, we demonstrate that the liquid droplets, which are stabilized by electrostatic interactions, appear only in the process of toxic (i)-amyloid formation, but not in the less-toxic (r)-amyloid formation process. The effect of various additives such as arginine, 1,6-hexanediol, and salts on amyloid formation was also examined to investigate interactions that are important for amyloid formation. Our results indicate that the maturation processes of these two amyloids were significantly different, whereas the nucleation by hydrophobic interactions was similar. These results also suggest the difference in the formation mechanism of two different insulin amyloids is attributed to the difference in the intermolecular interactions and could be correlated with the cytotoxicity.

## Introduction

Many neurodegenerative diseases, such as Alzheimer’s disease, Parkinson’s disease, and prion diseases, are associated with primary amyloidosis caused by the accumulation of soluble/insoluble protein aggregates and amyloid fibrils^1,2^. Reactive amyloidosis, on the other hand, occurs as a complication of inflammation or tissue degradation. Insulin ball is one of these complications. Insulin is a 51-residue peptide hormone that consists of two polypeptide chains, the A chain (21 residues) and the B chain (30 residues), linked by two disulfide bonds^3,4^. This hormone is important in controlling glucose metabolism and diabetes treatment. However, insulin ball, which is essentially consistent with insulin amyloid, occurs against clinical guidelines^5^. Previous reports showed that amyloidosis not only inhibits the absorption of insulin analogs but is also cytotoxic to the surrounding tissue^6–8^. Therefore, the mechanism of the formation of insulin amyloids and their cytotoxicity is now attracting attention.

Our previous studies have shown that bovine insulin and human insulin form two types of amyloids under different conditions: a toxic amyloid from intact insulin ((i)-amyloid) and less toxic amyloid formed in the presence of the reducing reagent, Tris (2-carboxyethyl) phosphine hydrochloride (TCEP)((r)-amyloid)^9,10^. It was also revealed that this polymorphism of insulin amyloids could be recognized by luminescent conjugated oligothiophenes such as pFTAA and BTD21^11^. Importantly, insulin preparations used for diabetes treatment also form these two types of amyloids without TCEP^11^. Since two types of amyloids with different toxicities are formed from the same protein, insulin can be a good model to understand amyloid toxicity. However, the differences between (i)-amyloid and (r)-amyloid in the formation mechanism and cytotoxicity expression remain unclear.

This study investigated the mechanisms underlying the formation of (i)-amyloid and (r)-amyloid. First, we focus on the possibility of liquid–liquid phase separation (LLPS) based on previous observations^12^. LLPS leads to the formation of distinct liquid droplets within a coexisting liquid milieu. This process is driven by temporary interactions, such as electrostatic, hydrophobic, hydrogen bonding, π-π, and cation-π interactions. It has been reported that LLPS occurs during the formation of amyloid aggregates of various proteins, such as α-synuclein^13^, tau^14^, islet amyloid polypeptide^15^, and TDP-43^16,17^*.* The amyloid formation process in the droplet (e.g., nucleation and maturation) differs depending on the protein. A high concentration of amyloidogenic peptides in the liquid droplets might promote the formation of amyloid fibrils^18,19^. Herein, we investigated whether droplet or phase separation was involved in the formation of human insulin amyloid using optical microscopy, fluorescence microscopy, and fluorescence recovery after photobleaching (FRAP), which revealed the movement of fluorescence probes within droplets. We also evaluated the effect of 1,6-hexanediol (1,6-HD), which interferes with weak hydrophobic interactions and has the property of dissolving droplets^20–24^ and arginine (Arg), which inhibits hydrophobic interactions via cation-π interaction^25–30^. Anions, which modulate electrostatic interactions, were also used to evaluate the intermolecular interactions of proteins.

Our results indicate that the maturation process between (i)-amyloid and (r)-amyloid was significantly different, whereas the nucleation of (i)-amyloid and (r)-amyloid by hydrophobic interactions were similar. The droplets, which are stabilized by electrostatic interactions, appeared in the process of (i)-amyloid formation. This suggests that LLPS can be related to toxic amyloid formation. In contrast, (r)-amyloid formation was unaffected by droplets and electrostatic interactions. Our results shed new light on the relationship of the important interactions for the formation process of toxic amyloid and LLPS/droplets.

## Result

### Dynamics of insulin amyloids by fluorescence recovery after photobleaching (FRAP)

**Figure 1:**
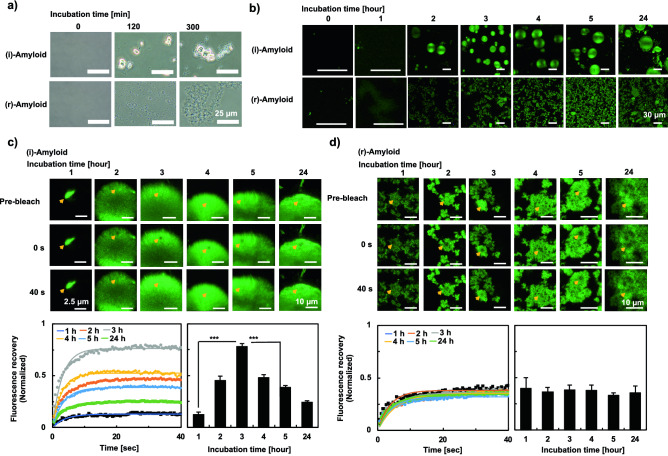
Morphology observation and evaluation of the liquidity of insulin amyloids. (**a**) Images of insulin amyloids were observed using an optical microscope. Insulin monomer was incubated for the indicated periods ((i)-amyloids, upper; (r)-amyloids, lower). (**b**) Fluorescence images showing the growth of droplets. (**c** and **d**) A representative spot at the indicated time points (upper) and FRAP measurements of insulin (i)-amyloids (**c**) and (r)-amyloids (**d**) to measure the change in fluorescence recovery (1 h (black), 2 h (red), 3 h (blue), 4 h (green) 5 h (orange), and 24 h (cyan) (lower left)) using the NIS-Elements (Ver. 5.21, Nikon) software. The intensities were normalized to the fluorescence intensity before bleaching. The orange arrows represent bleaching area. The lower right panels are the plots of normalized intensities before bleaching. Data represent the mean ± s.e.m. for 5 independent experiments. ***P < 0.005 (two-tailed Student’s *t*-test).

We first observed two types of human insulin amyloids using an optical microscope (Fig. 1a). The (i)-amyloid and (r)-amyloid samples were prepared as previously described^9,10^. Briefly, human insulin was incubated at pH1.6, 65 °C without ((i)-amyloid) or with ((r)-amyloid) a reducing reagent, TCEP. Interestingly, in the case of (i)-amyloid, droplets of several micrometers in size were observed 2 h after incubation. In contrast, droplets were not observed for (r)-amyloid, but amorphous agglomerate was formed after 5 h of incubation. These results suggest the formation of droplets by (i)-amyloid and differences in the amyloid formation mechanism between (i)-amyloid and (r)-amyloid.

Next, we further investigated the macroscopic structural difference between (i)-amyloids and (r)-amyloids and the liquidity of droplets using a fluorescence microscope and FRAP. Samples of human insulin amyloids were taken every hour. To obtain the structure and shape of droplets and aggregates, we used a solvatochromic push–pull pyrene dye, PK (1-acetyl-6-piperidyl pyrene), as a polarity sensitive probe with high brightness and photostability advantageous for microscopic applications^31^. PK is known to exhibit turn-on fluorescence in the presence of biological samples such as lipid membranes and droplets upon binding to their hydrophobic (i.e. lower polarity) site, and therefore expected to monitor the growth of insulin amyloids^31–33^. As shown in Fig. 1b, droplets were observed in the process of (i)-amyloid formation. This result agrees with the results shown in Fig. 1a. It was also shown that the fluorescence intensity peaked at 3 h and then decreased (Fig. S1). These results suggest that the amount of PK bound to hydrophobic surfaces increased due to (i)-amyloid formation and decreased due to the suppression of the influx of PK by the transition of droplets to solid-state. This result agrees with previous reports showing the transition to the solid-state of amyloid droplets formed by α-synuclein and FUS^13,34^. In contrast, droplets were not observed during (r)-amyloid formation, and the aggregate size and fluorescence intensity increased in a time-dependent manner (Fig. 1a,b, and Fig. S1), which indicates an increase in the hydrophobic surface. Interestingly, similar fluorescence images were obtained using thioflavin T (ThT) (Fig. S2), supporting that the droplets are filled with amyloids^35^. It is plausible that PK could bind to the insulin amyloid in the droplet since pyrene and its derivatives were shown to bind to various amyloids with hydrophobic interactions^36,37^.

Figure 1c ((i)-amyloid) and Fig. 1d ((r)-amyloid) show FRAP results during the formation of insulin amyloids. Notably, the fluorescence recovery rate increased with the incubation time from 1 to 3 h, reaching a maximum of about 0.8 (Fig. 1c). This result of (i)-amyloids indicates that the inside of the droplet during (i)-amyloid formation was highly fluid, supporting the droplet formation. Then, the fluorescence recovery rate decreased, with a recovery rate of about 0.2, indicating that the droplet is less fluid, possibly due to the suppression of Brownian motion by the formation of insoluble aggregates. This decrease is consistent with the observation of fibrillar amyloid aggregates after 24 h. On the other hand, the fluorescence recovery rate of (r)-amyloid was significantly small, suggesting that droplet was not formed during (r)-amyloid formation (Fig. 1d). It is noted that the fluorescence recovery rate remained 0.4 regardless of the incubation time. It is plausible that the inflow into the bleach region is due to the Brownian motion of PK. Importantly, these results suggest differences in the formation mechanism of (i)-amyloid and (r)-amyloid.

### The formation of insulin amyloids was inhibited by arginine and 1,6-hexanediol as additives

**Figure 2:**
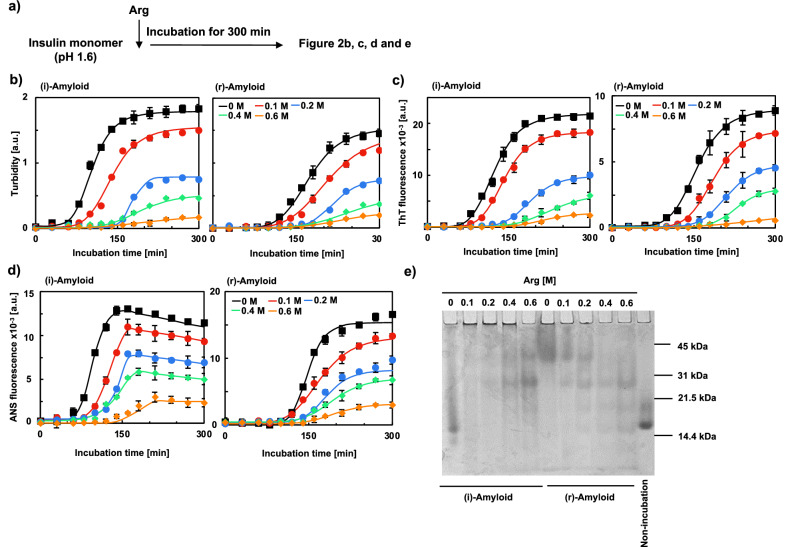
Evaluation of the effect of arginine on the formation of insulin amyloids. (**a**) Experimental scheme of (**b–e**). (i)-amyloid (left) and (r)-amyloid (right) were formed in the absence and presence of 0.1, 0.2, 0.4, 0.6 M Arg (black, red, blue, green, and orange, respectively), and were evaluated by Turbidity (**b**), ThT (**c**) and ANS (**d**) fluorescence intensity. Graphs of (**c**) and (**d**) are fluorescence values in emission wavelength (ThT, 490 nm; ANS, 480 nm). Protein concentrations were adjusted to 5 µM. (**e**) Native-PAGE analysis of 150 μM (i)-amyloids and (r)-amyloids formed in the absence and presence of 0.1, 0.2, 0.4, 0.6 M Arg. The rightmost sample is an unincubated native insulin.

As shown in Fig. 1, (i)-amyloid and (r)-amyloid showed differences in macroscopic appearance. We hypothesized that this difference was caused by differences in the intermolecular interactions in the aggregation process. To test this hypothesis, we first evaluated the effects of hydrophobic interactions on the formation of insulin amyloids using Arg as an additive (Fig. 2a). Arg is positively charged and binds to the protein surface through cation-π interactions with hydrophobic sites, including aromatic amino acids^38–40^. Eventually, the positive charge on the protein surface enhances the repulsion between proteins and inhibits hydrophobic interactions^25–30^. Figure 2b shows the change in turbidity during the formation of insulin amyloids in the absence and presence of Arg at various concentrations (0, 0.1, 0.2, 0.4, and 0.6 M). The aggregation of (i)-amyloid and (r)-amyloid was inhibited in an Arg concentration-dependent manner. This result indicates that cation-π interactions between Arg and hydrophobic sites inhibit insulin amyloid formation, indirectly suggesting that hydrophobic interactions are important in forming the two types of amyloids. Similar results were obtained by the evaluation of insulin amyloids using the ThT assay (Fig. 2c). These results were supported by the scanning electron microscopy (SEM) and transmission electron microscopy (TEM) observation showing suppression of amyloid formation by 0.6 M Arg, while insulin amyloids were formed in the absence of Arg (Figs. S3 and S4). It should be noted that the increasing tendency of turbidity is consistent with that of ThT fluorescence intensity. Therefore, it is plausible that the increase in the accumulated β-sheet structure coincides with the increase in aggregates. It was also demonstrated that the lag time of insulin amyloids incubated with Arg increased dose-dependently, suggesting that Arg inhibited the nucleation of insulin amyloids. We also evaluated the amount of exposed hydrophobic surface by the measurement of the 1-anilinonaphthalene 8-sulfonate (ANS) fluorescence intensity (Fig. 2d). The time-dependent increase in ANS fluorescence intensity suggests that the exposed hydrophobic sites increase with the growth of amyloid. Moreover, the ANS fluorescence intensity of (i)-amyloid samples at 150 min was higher than that of mature (i)-amyloid. This high intensity may suggest the formation of insulin oligomers since previous study has shown that the ANS fluorescence intensity of Aβ42 oligomers is higher than that of mature fibrils^41^. The oligomer formation is supported by the results of native-PAGE, showing that some multimeric bands were observed, and the molecular weights increased in an Arg concentration-dependent manner (Fig. 2e). This result is consistent with previous studies showing that the nucleus of insulin amyloid is formed stepwise from dimer → two dimer units → three dimer units^42–44^. It is noted that the change of ANS fluorescence intensity of the samples without Arg was similar to that of PK (Fig. S1), supporting that PK could recognize hydrophobic sites. Collectively, the increase of lag time and the decrease of aggregates and the exposed hydrophobic sites by Arg were observed both in (i)-amyloid and (r)-amyloid, suggesting that the nucleation processes of both (i)-amyloid and (r)-amyloid were similar and hydrophobic interactions play a major role in both processes.

**Figure 3:**
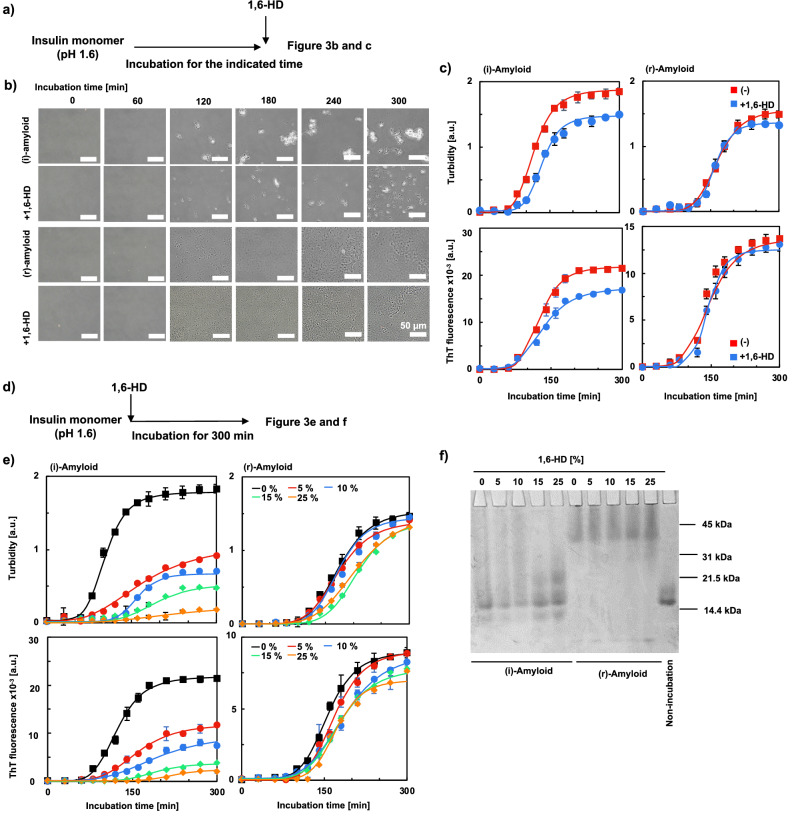
Evaluation of the effect of 1,6-HD on the formation of insulin amyloids. (**a**) Experimental scheme of (**b**) and (**c**). (**b**) Images of insulin amyloids were observed using an optical microscope. Insulin was incubated for the indicated periods ((i)-amyloids, top; (r)-amyloids, third from the top), were observed after the additives of 5% 1,6-HD ((i)-amyloids: second from the top, (r)-amyloids: fourth from the top). The scale bar is 50 μm. (**c**) Turbidity (upper) and ThT assay (lower) of (i)-amyloid (left) and (r)-amyloid (right)(red) measured after adding 5% 1,6-HD (blue). Peak fluorescence values at 490 nm (ThT). Protein concentrations were adjusted to 5 µM. (**d**) Experimental scheme of (**e**) and (**f**). (**e**) (i)-amyloid (left) and (r)-amyloid (right) were formed in the absence (black) and presence of 5, 10, 15, 25% 1,6-HD (red, blue, green, and orange, respectively), and were evaluated by turbidity (upper) and ThT assay (lower). Peak fluorescence values at 490 nm (ThT). Protein concentrations were adjusted to 5 µM. (**f**) Native-PAGE analysis of 150 μM (i)-amyloids and (r)-amyloids formed in the absence and presence of 5, 10, 15, 25% 1,6-HD. The rightmost sample is an unincubated native insulin.

The effect of 1,6-HD on insulin amyloid formation was then investigated (Fig. 3a). Figure 3b shows the optical microscopic images of insulin amyloid samples incubated for the indicated periods and those taken soon after the addition of 5% 1,6-HD. Circular structures of several micrometers were observed after 120 min incubation of (i)-amyloid, which is consistent with the results shown in Fig. 1. Interestingly, (i)-amyloid aggregates were dissolved by the addition of 1,6-HD. This result suggests that (i)-amyloid has the characteristics of liquid droplets since 1,6-HD was known to dissolve droplets^20–24^. This droplet formation is supported by the results obtained by FRAP, which showed high fluidity inside the droplet. These results also suggest that the formation of (i)-amyloid is associated with weak hydrophobic interactions, considering the property of 1,6-HD that disrupts weak hydrophobic interactions^20–24^. The decrease in the droplet by the addition of 1,6-HD was supported by the measurement of turbidity and ThT fluorescence intensity (Fig. 3c). In contrast, in the (r)-amyloid sample, no significant change in the microscopic images was observed for the incubated (r)-amyloid with the addition of 1,6-HD (Fig. 3b). This result was supported by the results of turbidity and ThT assays (Fig. 3c). Taken together with the results of ANS assay above (Fig. 2d), it is plausible that strong hydrophobic interaction in (r)-amyloid was not affected by 1,6-HD.

Furthermore, we evaluated insulin amyloid samples incubated in the presence of 1,6-HD at various concentrations (0, 5, 10, 15, and 25%) for 300 min (Fig. 3d). The turbidity and ThT fluorescence intensity of (i)-amyloid samples decreased in a 1,6-HD concentration-dependent manner, whereas (r)-amyloid samples showed no significant decrease, indicating that the prevention effect of 1,6-HD is not on (r)-amyloid formation but on (i)-amyloid formation (Fig. 3e). This result was supported by the SEM and TEM observations (Figs. S3 and S4). The molecular weight of insulin samples incubated in the absence or presence of 1,6-HD at various concentrations was analyzed by native-PAGE (Fig. 3f). As shown in the figure, low-molecular-weight multimer increased in 1,6-HD concentration-manner for the (i)-amyloid samples, whereas (r)-amyloid samples showed no significant change. Thus, it is plausible that 1,6-HD affected (i)-amyloids by hydrophobic interactions because the insulin amyloid has a hydrophobic accumulated β-sheet structure^45^, and hence prevents amyloid formation. On the other hand, 1,6-HD was less effective in (r)-amyloid formation, and this result is similar to the result shown in Fig. 3b,c.

**Figure 4:**
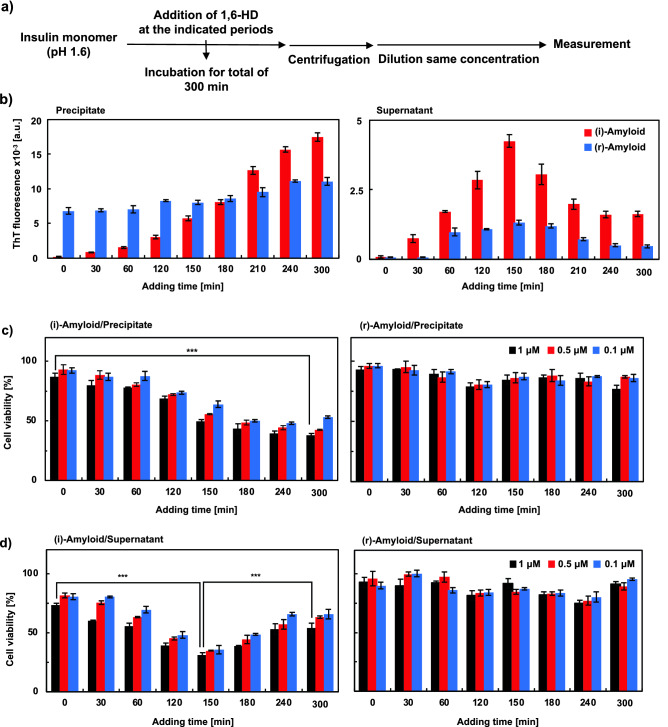
Evaluation of effect of 1,6-HD added on the formation process of the insulin amyloids. (**a**) Experimental scheme of (**b**–**d**). Samples in the precipitate (left) and the supernatant (right) are (i)-amyloids (red) and (r)-amyloids (blue) with 25% 1,6-HD added at the indicated time points and incubated for a total of 300 min. These samples were centrifuged at 15,000 rpm for 15 min and evaluated using ThT (**b**). Peak fluorescence values at 490 nm (ThT). Protein concentrations were adjusted to 5 µM. (**c** and **d**) Cytotoxicity of the samples in the precipitate (**c**) and the supernatant (**d**) of (i)-amyloids (left) and (r)-amyloids (right) with 25% 1,6-HD added at the indicated time points and incubated for a total of 300 min. These samples were centrifuged at 15,000 rpm for 15 min, and cytotoxicity was evaluated using the MTT assay against HeLa cells. All samples were quantified by BCA assay and were diluted to the same protein concentration: 1, 0.5, and 0.1 μM (black, red, and blue, respectively). The absorbance was normalized to 100% in PBS. ***P < 0.005 (two-tailed Student’s *t*-test).

To further investigate the effect of 1,6-HD on insulin amyloid formation, 25% 1,6-HD was added at various incubation times and subsequently incubated for a total of 300 min (Fig. 4a). Figure 4b shows the ThT fluorescence intensity of the samples, which were separated into precipitate and supernatant by centrifugation. It should be noted that the same protein concentration (5 µM) was used in this assay. ThT fluorescence intensities of (i)-amyloid samples in the precipitates were relatively low when 1,6-HD was added within 60 min of incubation and then increased in a time-dependent manner. This result indicates that (i)-amyloid formation was suppressed by adding 1,6-HD during the lag time of (i)-amyloid. As shown in Figure S5, SEM observation of (i)-amyloid with 1,6-HD, 150 min after the incubation showed fibrillar structures, whereas (i)-amyloid added with 1,6-HD during the lag time showed the inhibition of aggregation.

Similar results were obtained for (i)-amyloid samples containing Arg instead of 1,6-HD during the lag time (Fig. S6a). This result may be due to hydrophobic interactions inhibition by both Arg and 1,6-HD^20,21^. However, the precipitates containing 1,6-HD or Arg, 150 min after incubation, showed a difference in the effect between 1,6-HD and Arg on (i)-amyloid formation. The inhibition effect by 1,6-HD was approximately 65% at 150 min, whereas the effect by Arg was approximately 33% when compared with the values at 300 min, indicating that the effect of Arg was smaller than that of 1,6-HD (Fig. S7a). This difference might be due to the difference in the interaction mechanism of Arg and 1,6-HD. Arg interacts with aromatic amino acid residues via cation-π interaction to block hydrophobic interactions^25–30^, whereas 1,6-HD is considered to inhibit weak hydrophobic interactions as an aliphatic alcohol^20–24^. It is also plausible that the maturation of (i)-amyloid is inhibited by the dissolution of droplets as a property of 1,6-HD, whereas the disruption of hydrophobic interactions by Arg is not enough to inhibit the further aggregation of (i)-amyloid. Moreover, difference in the effect of temporal increases of ThT intensities of the supernatant samples between 1,6-HD and Arg was observed. The peak ThT intensity with the addition of 1,6-HD at 150 min was approximately 160% higher than the ThT intensity at 300 min, whereas that with Arg was approximately 50% higher than the ThT intensity at 300 min (Fig. S7b).

In the case of the (r)-amyloid samples, the ThT fluorescence intensities of the precipitate formed with 1,6-HD did not change compared with that of (i)-amyloid (Fig. 4b left). The changes of the fluorescence intensities of the supernatant samples of (r)-amyloid were also not significant considering the intensity of the mature (r)-amyloid (Fig. 4b right). On the other hand, ThT fluorescence intensities of the precipitate of (r)-amyloid formed in the presence of Arg instead of 1,6-HD were relatively low when Arg was added within 120 min of incubation and then gradually increased (Fig. S6a). SEM observation of (r)-amyloid to which 1,6-HD was added showed that 1,6-HD was less effective in the formation of (r)-amyloid, which was consistent with the results of ThT fluorescence intensity (Fig. S5). This is caused by the suppression of nucleation within the lag time by inhibiting hydrophobic interactions as discussed above (Fig. 2).

Next, we investigated the cytotoxicity of these insulin samples to which 25% 1,6-HD was added during the formation of insulin amyloids against HeLa cells using the MTT assay. The cytotoxicity of both the precipitate (Fig. 4c) and the supernatant (Fig. 4d) after centrifugation was evaluated. The (i)-amyloid samples to which 1,6-HD was added within 60 min of incubation were non-toxic, consistent with the suppression of aggregation. The cell viabilities of (i)-amyloid samples in the precipitates then decreased in adding time of 1,6-HD dependent manner, possibly due to maturation into insoluble species. Importantly, the highest cytotoxicity of the supernatant samples of (i)-amyloid was observed for the samples treated with 1,6-HD 150 min after incubation (Fig. 4d). This may be due to the presence of soluble toxic species as discussed below. Then, the cytotoxicity was reduced, and the cell viability was restored to 70%. This result indicates that toxic species in the supernatant reduced and matured into insoluble (i)-amyloid. It was also shown that soluble toxic species were retained due to the dissolution of droplets by 1,6-HD. The cell viabilities of (i)-amyloid samples in the precipitates also decreased by the addition of Arg (Fig. S6b). On the other hand, the decrease in cell viability of the supernatant of (i)-amyloid samples incubated with Arg was not significant (Fig. S6c). This result suggests that oligomeric species are not present in the supernatant because of subsequent aggregation despite the addition of Arg. In contrast, the cell viabilities of all the (r)-amyloid samples were close to 80–100%, possibly because (r)-amyloid was non-toxic itself. Cell viability was also examined by LDH assay and Caspase-3 activity assay (Figs. S8 and S9). LDH release was increased for the (i)-amyloid samples to which 0.6 M Arg or 25% 1,6-HD were added, which showed reduced cell viability by MTT assay (Fig. 4c,d). Caspase-3 activity was also increased for these insulin amyloid samples. These results suggest that cell death was induced by apoptosis. These results are consistent with the previous studies showing that apoptosis was induced by soluble species of various amyloidogenic proteins such as amylin, Aβ, and TDP43^46–48^.

To examine the surface properties of the soluble toxic species, the surface hydrophobicity of these samples was evaluated by ANS assay (Fig. S10). ANS fluorescence intensities of the precipitated (i)-amyloid samples incubated with 25% 1,6-HD and 0.6 M Arg increased in an adding time-dependent manner (Fig. S10a and b, left panels). This result indicates that the additives inhibit the further increase of the exposed hydrophobic surface of the insoluble aggregates. Interestingly, the supernatant sample of (i)-amyloid to which 1,6-HD was added at 150 min of incubation time, which was the most cytotoxic, had the highest ANS fluorescence intensity (Fig. S10a, right panel). This result suggests that the high ANS intensity is correlated with oligomer formation and that the soluble toxic species is preserved by 1,6-HD. In contrast, the ANS fluorescence intensity of the supernatant sample of (i)-amyloid increased for the samples to which Arg was added at 0–150 min of incubation times, but no significant change was observed for the samples to which Arg was added at incubation times longer than 150 min (Fig. S10b, right panel). This result suggests that Arg could inhibit the nucleation process by hydrophobic interaction, consistent with the results shown in Fig. 2, but the inhibition of hydrophobic interactions by Arg could not suppress the aggregation of (i)-amyloid during the maturation process. Since it was shown that droplet of (i)-amyloid was formed after incubation around 150 min (Figs. 1 and 3), it was suggested that the toxic oligomers were formed by dissolution of droplet by 1,6-HD, not by the inhibition of hydrophobic interaction by Arg.

The surface properties of the samples used in Fig. 4 were also evaluated with a dot blot assay using the oligomer-specific A11 polyclonal antibody, which has been widely used in the specific detection of the formation of oligomeric species^49^ (Fig. S11). The (i)-amyloid samples to which 25% 1,6-HD was added at 0 or 60 min of incubation were not recognized by the A11 antibody (Fig. S11a). This agrees with the results showing that aggregation was inhibited under these conditions (Fig. 4b and Fig. S6). Interestingly, the (i)-amyloid samples for which 1,6-HD was added at 150 min after incubation times showed high dot intensities in both the un-centrifuged sample and supernatant sample, indicating that the soluble toxic species have an A11-positive oligomeric structure by dissolution effect of the droplet. Importantly, this result also suggests that the oligomeric structure is preserved because of the inhibition of maturation by the addition of 1,6-HD. When the (i)-amyloid sample was incubated with 0.6 M Arg, the A11-positive oligomeric structure was formed for the samples to which Arg was added at 150 min of incubation time (Fig. S11b). In contrast with the result of 1,6-HD, the oligomeric structure was not preserved since the intensity was similar to that of the mature (i)-amyloid, suggesting that Arg could not inhibit the maturation process of (i)-amyloid (Fig. S11b). This result is also consistent with the result obtained by the ANS assay (Fig. S10b).

### Effect of Hofmeister series and electrostatic shielding on the formation of insulin amyloids assessed by salts

**Figure 5:**
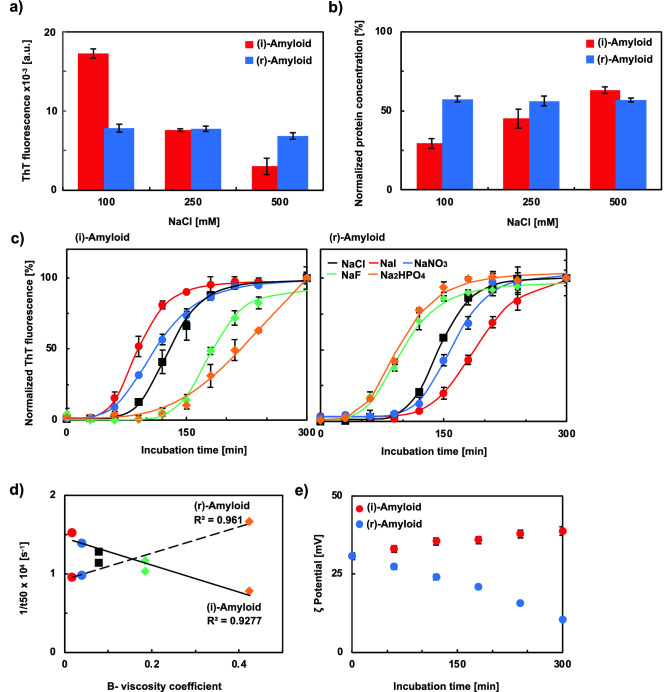
Evaluation of the effect of salts on the formation of insulin amyloids. (**a**) ThT assay to confirm the dependency of the NaCl concentration: (i)-amyloids (red) and (r)-amyloids (blue). Peak fluorescence values at 490 nm (ThT). Protein concentrations were adjusted to 5 µM. (**b**) BCA assay of supernatant samples to confirm the dependency of the NaCl concentration: (i)-amyloids (red) and (r)-amyloids (blue). Protein concentrations were normalized to 2 mg/mL. (**c**) ThT assay to confirm the effect of salts on the kinetics of insulin amyloid formation of (i)-amyloids (left) and (r)-amyloids (right) using the Hofmeister series of salts (100 mM, NaI, red; NaNO_3_, blue; NaCl, black; NaF, green; Na_2_HPO_4_, orange). Normalized peak fluorescence values at 490 nm (ThT) are shown. Protein concentrations were adjusted to 5 µM. (**d**) The rank order of the effects of different anions on the rate (1/t_50_) of insulin amyloids formation at 100 mM added salt versus their order in the Jones–Dole B-coefficient of the Hofmeister series of salts (NaI, red; NaNO_3_, blue; NaCl, black; NaF, green; Na_2_HPO_4_, orange). The approximation straight lines for (i)-amyloid (solid line) and (r)-amyloid (dotted line) are shown. (**e**) ζ potential measurement to confirm the time dependency of the charge: (i)-amyloids (red) and (r)-amyloids (blue). These samples were incubated for the indicated periods. The protein concentration in the precipitate was adjusted 86 µM (0.5 mg/mL). The average values of three repeated measurements are shown.

Previous research has shown that droplets formed during the formation of amyloids are stabilized by electrostatic interactions^14,18,50^. Thus, it is necessary to investigate the relationship between the formation of insulin amyloids and electrostatic interactions. We evaluated the effect of NaCl on the formation of insulin amyloids using the ThT and BCA assays. As shown in Fig. 5a, ThT fluorescence intensities of (i)-amyloid samples decreased in a NaCl concentration-dependent manner, possibly because the electrostatic interaction is inhibited by the increase in the electrostatic shielding effect with NaCl concentration. In contrast, a decrease in ThT fluorescence intensities of (r)-amyloid samples was not observed. The protein concentration in the supernatant after centrifugation was quantified to confirm the decrease in aggregates by NaCl (Fig. 5b). The results indicate that the amount of protein in the supernatant of the (i)-amyloid samples increase in a NaCl concentration-dependent manner, indicating that the amounts of aggregates decrease. These results suggest that electrostatic interactions are involved in (i)-amyloid but not (r)-amyloid formation.

Next, we investigated the correlations between the formation of insulin amyloids and the Hofmeister series. The Hofmeister series is a parameter that indicates the relative influence of ions on proteins in terms of solubility and stability. Kosmotropic ions (typically NaF and Na_2_HPO_4_) have the salting-out effect that decreases the solubility of solutes, leading to stabilization and the compact structure of proteins; conversely, chaotropic ions (typically NaI) has the salting-in effect of solutes that increase the solubility of proteins^51,52^. Two types of insulin amyloids, (i)-amyloid and (r)-amyloid, were formed in the presence of 100 mM NaI, NaNO_3_, NaCl, NaF, or Na_2_HPO_4_, and were evaluated by measuring ThT fluorescence intensity (Fig. 5c). Figure 5d shows the correlation between the rate of amyloid formation and the relative viscosity of each salt. The 1/t_50_ [s^-1^] values, which indicate the reciprocal of t_50_, the time point at which the amyloid formation is 50% complete, obtained from Fig. 5c, were used to determine the rate of amyloid formation^53^. The B-viscosity coefficient values of the Jones-Dole equation, which expresses the relative viscosity of salt solutions as a function of salt concentration, were used for the relative viscosity^54,55^. The order of anions that induce amyloid formation under these conditions was: (i)-amyloid, I^-^ > NO_3_^-^ > Cl^-^ > F^-^ > HPO_4_^2-^; (r)-amyloid, HPO_4_^2-^ > F^-^ > Cl^-^ > NO_3_^-^ > I^-^. These results suggest that (i)-amyloid formation is accelerated by chaotropic anions, while (r)-amyloid formation is promoted by kosmotropic ions. This difference would suggest a difference in affinity for anions between (i)-amyloids and (r)-amyloids. It should be noticed that the amyloid morphology was similar for the insulin amyloids formed with kosmotropic ions and chaotropic ions (Fig. S12). To investigate the cause of the difference in the affinity, the zeta potential of the insoluble aggregate samples of (i)-amyloid and (r)-amyloid was measured (Fig. 5e). The positive charge quantity of insoluble aggregates of (i)-amyloid increased, while that of insoluble aggregates of (r)-amyloid decreased in an incubation time-dependent manner. This result suggests that the surface charge of insoluble aggregates would cause a difference in the type of anion with a high affinity for each insulin amyloid.

## Discussion

**Figure 6:**
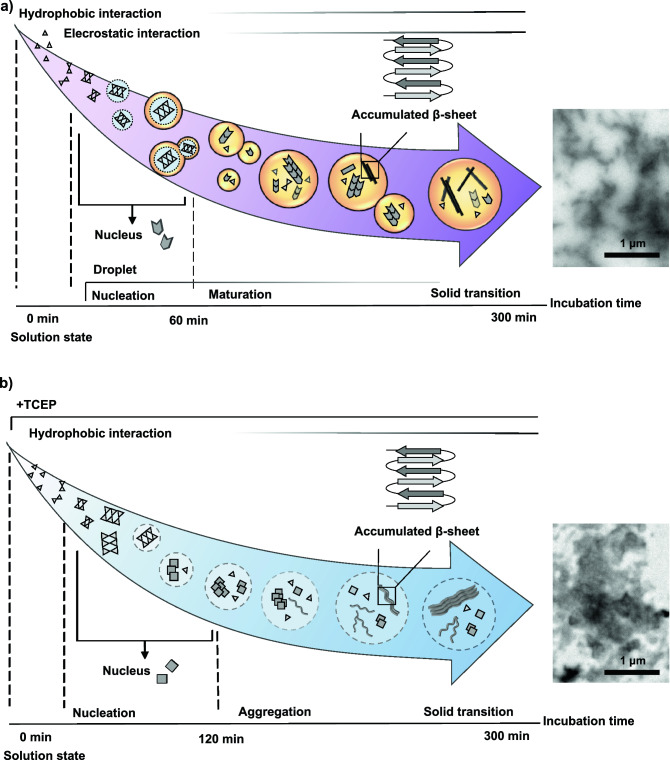
Scheme of the proposed mechanism for the formation of insulin (i)-amyloids (**a**) and (r)-amyloids (**b**). (**a**) The nucleuses of (i)-amyloid (hexagon) form insulin monomer (triangle). The droplets (orange circle) are formed in the elongation reaction (rectangle), and the maturation process proceeds in the droplets. (**b**) The nucleuses of (r)-amyloid (square) form insulin monomer (triangle). The maturation process of (r)-amyloid is dependent on incubation time (dotted circles).

In this study, the difference in the formation mechanism of insulin amyloids, toxic (i)-amyloid and non-toxic (r)-amyloid, was investigated as model amyloid to understand the possible correlation between cytotoxicity and the formation mechanism. Based on our results, we propose a schematic representation of the mechanism of (i)-amyloid and (r)-amyloid (Fig. 6). The importance of hydrophobic interactions was demonstrated by evaluating the effect of Arg, which is considered to inhibit hydrophobic interactions through cation-π interactions^25–30^, on the formation of (i)-amyloid and (r)-amyloid. Our results also showed that the addition of Arg inhibited the nucleation process of (i)-amyloid and (r)-amyloid. These results indicate the importance of hydrophobic interactions in the nucleation of both types of insulin amyloids, which is consistent with previous reports^56^. Interestingly, a stepwise polymerization reaction of insulin monomer was observed, suggesting that the important interactions in the nucleation process of these amyloids were similar.

Importantly, droplet-like structures, which were filled with accumulated β-sheet structures and had high liquidity was observed in the process of (i)-amyloid formation, but not in the process of (r)-amyloid formation. This result suggests that droplet formation may be correlated with amyloid cytotoxicity. The fluorescence intensity of PK/ANS of (r)-amyloid increased in an incubation time-dependent manner, possibly due to strong hydrophobic interaction in (r)-amyloid formation. These results also suggest a difference in the aggregation process between (i)-amyloid and (r)-amyloid. The droplets formation of (i)-amyloid was supported by the dissolution of the droplet by 1,6-HD, which has been known to possess the properties of the inhibition of weak hydrophobic interactions and the dissolution of the droplet^20–24^. Interestingly, the formation of (r)-amyloid was unaffected by 1,6-HD, possibly because the hydrophobic interactions acting in the formation process of (r)-amyloid are stronger than those that are inhibited by1,6-HD. Furthermore, the addition of 1,6-HD during the forming (i)-amyloid inhibited maturation and retained highly toxic oligomers in the supernatant. Therefore, it was suggested that droplets also play an important role in the maturation process of (i)-amyloid. The dissolution of droplets suppresses the growth of toxic oligomers. This is consistent with the difference in cytotoxicity between (i)-amyloid and (r)-amyloid^9,10^, suggesting that the maturation process is significantly different between (i)-amyloid and (r)-amyloid.

We also evaluated the effect of various salts on insulin amyloid formation to investigate the role of electrostatic interactions. The result using NaCl suggests that electrostatic interactions are involved in (i)-amyloid, but not (r)-amyloid formation. This result agrees with previous reports that electrostatic interactions contribute to the stabilization of the droplets^14,18,39,50^. Interestingly, (i)-amyloid formation was promoted by chaotropic ions, while (r)-amyloid was promoted by kosmotropic ions. The promotion effect of (i)-amyloid by chaotropic ions follows the inverse Hofmeister series. Previous studies have shown that chaotropic anions promote aggregation by destabilizing proteins^54,55,57^. Thus, it is considered that the (i)-amyloid formation is accelerated by the destabilizing effect of chaotropic anions. The insoluble products of (r)-amyloid have a lower affinity for anions than those of (i)-amyloid and caused the salting-out effect by increasing surface tension. The kosmotropic anions induced salting-out and promoted (r)-amyloid formation^58,59^. This tendency is consistent with previous research showing that the aggregation of TDP-43 is related to LLPS and is promoted by the inverse Hofmeister series^17^. Therefore, a possible relationship between the inverse Hofmeister series and the occurrence of droplets/LLPS can also be suggested.

In summary, this study demonstrated the difference in the formation mechanisms of (i)-amyloid and (r)-amyloid. The similar nucleation processes of (i)-amyloid and (r)-amyloid are affected by hydrophobic interactions. Droplets stabilized by electrostatic interactions work during the maturation process of (i)-amyloid, whereas (r)-amyloid is formed by strong hydrophobic interactions. Although those experiments were carried out at unphysiological conditions (65 °C and pH 1.6), it is noted that droplet was also observed for (i)-amyloid at physiological conditions (37 °C and pH 7.4) (Fig. S13a), and (i)-amyloid formation was inhibited by the suppression of electrostatic interactions (Fig. S13b), supporting a physiological relevance of this study. Although future research should be carried out to investigate a more detailed mechanism of the formation of insulin amyloids from insulin preparations and the exhibition of cytotoxicity, these insights into the difference between the formation mechanism of two different insulin amyloids and their interactions may shed new light on clarifying the common underlying mechanism of the amyloid formation and the development of cytotoxicity.

## Materials and methods

### Materials

Human insulin was purchased from Wako (Fujifilm, Tokyo, Japan). Tris (2-carboxyethyl) phosphine hydrochloride (TCEP) was obtained from Sigma-Aldrich (St. Louis, MO, USA). 1,6-hexanediol (1,6-HD), (L)-arginine (Arg), and Ficoll(R) 400 were purchased from Wako (Tokyo, Japan). PK was prepared as previously described^32^. All other reagents were of analytical grade. Aqueous solutions were prepared using deionized Milli-Q water (Millipore, Billerica, MA, USA).

### Preparation of insulin amyloids

2 mg/mL (344 µM) insulin solutions were freshly prepared for each (i)-amyloid formation experiment in 25 mM HCl, 100 mM NaCl, and adjusted to pH 1.6^43,60^. (r)-Amyloid formation was induced by incubating the insulin solution with 50 mM TCEP. The two types of insulin solution were incubated at 65 °C for 5 h without agitation. To prepare the precipitated and supernatant samples, insulin amyloids incubated were centrifuged at 15,000 rpm for 15 min. Protein concentrations in the supernatant were analyzed using a BCA protein assay kit (Thermo Fisher Scientific, Waltham, MA, USA). Precipitated and supernatant samples were diluted with PBS to equal the protein concentration of each sample. Insulin solutions with 10% Ficoll were incubated at 65 °C for 5 h without agitation (pH 1.6) to prepare the insulin amyloids for FRAP experiments.

To estimate the effect of additives on insulin amyloid formation, Arg and 1,6-HD were added to the insulin solutions at different time points at various concentrations (0–0.6 M Arg and 0–25% 1,6-HD). It was confirmed that pH of the solution was not changed by the addition of Arg and 1,6-HD at the examined concentrations (data not shown). To investigated the correlations between the formation of insulin amyloids and the Hofmeister series, (i)-amyloid and (r)-amyloid, were formed in the presence of 100 mM NaI, NaNO_3_, NaCl, NaF or Na_2_HPO_4_.

### Turbidity measurement

Turbidity measurements (optical density at 600 nm) were performed using a V-670 UV–VIS–NIR spectrophotometer (Jasco, Tokyo, Japan). Insulin samples (15 μL) were diluted in PBS to a final volume of 150 μL, and a final concentration of 0.2 mg/mL. The turbidity of the above samples was measured at 600 nm at 25 °C.

### Fluorescence measurement

Fluorescence spectra were measured at 25 °C with a microplate reader (Synergy H1, BioTek, Winooski, VT, USA). The fluorescence intensities of insulin amyloids were measured in the presence of ThT or ANS in PBS (pH 7.4) as described^10^. Each sample was prepared with 5 μM protein and 1 μM probe concentration. The excitation wavelength of ThT was set to 420 nm, and the emission was measured at 490 nm. The excitation wavelength of the ANS was set to 360 nm, and the emission was measured at 480 nm.

### Native polyacrylamide gel electrophoresis (native-PAGE)

Insulin samples (344 μM) were mixed with the native-PAGE sample buffer at 0.87 mg/mL final protein concentration. Insulin samples (15 μL) were mixed with 5 μL of native sample buffer and run on a 10% acrylamide gel under native conditions. Unstained protein standards (low range, BioRad, Hercules, CA, USA) and unincubated insulin were used to determine the apparent molecular weights of the peptides and aggregates.

### Scanning electron microscopy (SEM)

Insulin samples (5 μM) were dropped onto a silicon wafer and allowed to air-dry. The samples were observed at an acceleration voltage of 15 kV using a field-emission scanning electron microscope (FE-SEM, JSM7001FA, JEOL, Tokyo, Japan).

### Transmission electron microscope (TEM)

TEM measurements were performed using a JEM-1400 Flash transmission electron microscope (JEOL, Tokyo, Japan) operated at 120 kV. Samples were diluted with distilled water and negatively stained with 2% (w/v) uranyl acetate solution on copper grids (F-150 grid, Nisshin EM, Tokyo, Japan) covered by carbon-coated Formvar film.

### MTT assay

Cell viability was determined using an MTT cell proliferation kit (Roche, Basel, Switzerland), as previously described^10^. HeLa cells were maintained in EMEM medium with 10% fetal bovine serum, penicillin (100 U/mL), and streptomycin (100 mg/mL) in 5% CO_2_ at 37 °C. Cells were plated at a density of 25,000 cells/well in 96-well plates and grown overnight. Cells were subsequently incubated in the absence (control) and presence of insulin amyloids for 24 h. After 24 h of incubation, 10 µL of MTT reagent was added to each well, according to the manufacturer’s directions. The cells were then incubated for 4 h at 37 °C. The reaction was stopped by adding 100 µL of 10% SDS in 10 mM HCl. Plates were read at 562 nm using a microplate reader (BioTek). Each data point was the average of triplicates.

### LDH assay

We performed LDH assays using an LDH cytotoxicity detection kit (Takara-Bio, Tokyo, Japan) according to the manufacturer's instructions. HeLa cells were plated at a density of 25,000 cells/well in 96-well plates and grown overnight. Cells were subsequently incubated in the absence (negative control), and presence of 0.1% Tween20 (positive control) or insulin amyloid samples for 24 h. After 24 h of incubation, 100 µL of reagent mixture was added to each well. The plates were then incubated for 30 min at room temperature. Plates were read at 490 nm using a microplate reader (BioTek). Each data point was the average of triplicates.

### Caspase-3 activity assay

The caspase-3 activity assay was carried out using the caspase-3 fluorometric assay kit (Sigma-Aldrich) done according to the manufacturer’s instructions. HeLa cells were plated at a density of 25,000 cells/well in 96-well plates and grown overnight and were incubated with insulin amyloid samples for 24 h. After 24 h of incubation, cells were collected and lysed in cell lysis buffer, 200 µL of assay buffer was added to each well. The plates were then incubated for 60 min at 37 °C. Fluorescence was measured using a microplate reader (BioTeK, ex: 360 nm/em: 460 nm). Each data point was the average of triplicates.

### Dot blot

Insulin samples (2 μL) were spotted onto a nitrocellulose membrane (0.22 μm, GE Healthcare Life Sciences, Buckinghamshire, UK). The membrane was blocked with 5% skim milk in 5% BSA/Tris-buffered saline (TBS) containing 0.01% Tween 20 for 1 h at room temperature. Subsequently, the membrane was incubated with primary anti-insulin antibody (1:3000, ab181547, Abcam, Cambridge, MA, USA) and anti-oligomer antibody (1:3000, A11, Invitrogen, Carlsbad, CA, USA) for 24 h. The following day, the membrane was incubated with the secondary anti-rabbit IgG antibody (1:10,000; R&D Systems, Minneapolis, MN, USA) for 1 h, as described^10^. After washing with TBST, proteins were visualized using an ECL blotting detection kit (Bio-Rad) according to the manufacturer’s instructions. Luminescence was detected with a Las 4000 mini luminescent image analyzer (Fujifilm, Tokyo, Japan) using the Image Reader Las 4000 software. ImageJ was used to determine the intensity of each dot, which was used to quantify the amount of antibodies on the membrane.

### FRAP

FRAP experiments were carried out on particles of 2.0 μm side (0.5 μm for the (i)-Amyloid samples incubated for 1 h) by photobleaching a square region encompassing the droplet using a Nikon upright confocal microscope A1R (Tokyo, Japan) with a water immersion objective lens (Plan Apo IR 60 × WI DIC N2). Bleaching was performed using a 403 nm solid-state laser at 20% power for iterations at 0.237 s bleaching time per iteration to bleach the particle completely. The particle was imaged over time by a time series at no intervals, including 2.59 s before bleaching and up to 41.45 s after bleaching. All measurements were performed in quintuplicate using the NIS-Elements (Ver. 5.21, Nikon) software.

Fluorescence recovery curves were constructed from the total intensity fluorescence values in the unbleached regions of interest (ROIs) for each frame corrected for the background and laser scanning bleaching. To calculate the normalized fluorescence intensity, *I (n)*, the following equation was used:

$$I\left( n \right) = \frac{{\left[ {I\left( t \right) - I\left( m \right)} \right]}}{r}$$

*I (t)* = fluorescence intensity at time *t. I (m)* = minimum of fluorescence intensity. *r* = *I*_*c*_/*I*_*c*0_ rate of photo-bleaching. *I*_*c*_ = fluorescence intensity of the ROI after photo-bleaching. *I*_*c0*_ = fluorescence intensity of the ROI before photo-bleaching.

The normalized and background-corrected fluorescence recovery curves were fitted using a single exponential recovery function. The fitted equation is as follows:

$$I\left( t \right)\, = \,A \, \left( {1 - e^{ - kt} } \right)$$

*A* = mobile fraction. *k* = decay rate.

The half time of the recovery *(t*_*1/2*_*)* was calculated from^61^,

$$t_{1/2} = \frac{{{\text{ln}}\left( 2 \right)}}{k}$$

The diffusion coefficient *(D)* is calculated by the following equation^62^.

$$D = \frac{{0.244R^{2} }}{{t_{1/2} }}$$

*R* = radius of the bleached ROIs. *t*_*1/2*_ = half-time of the recovery.

## Supplementary Information

Supplementary Information.

## Data Availability

All data generated or analyzed during this study are included in this published article (and its Supplementary Information files).
