# Supplementary material for: Differences in interaction lead to the formation of different types of insulin amyloid
Source: Sci Rep. 2022 May 20;12:8556. doi: 10.1038/s41598-022-12212-6 (PMC9123177; doi:10.1038/s41598-022-12212-6)
Supplement: Supplementary file 1 — Supplementary Information. [file 41598_2022_12212_MOESM1_ESM.pdf]

Electronic Supplementary Information

Differences in interaction lead to the formation of different types of insulin amyloid

Wakako Mori[1], Ryosuke Kawakami[2], Yosuke Niko[3], Tomohiro Haruta[4], Takeshi Imamura[2], Kentaro Shiraki[5], and Tamotsu Zako[1]

[1] Department of Chemistry and Biology, Graduate School of Science and Engineering, Ehime University, Ehime, Japan

[2] Department of Molecular Medicine for Pathogenesis, Graduate School of Medicine, Ehime University, Ehime, Japan

[3] Research and Education Faculty, Multidisciplinary Science Cluster, Interdisciplinary Science Unit, Kochi University, Kochi, Japan.

[4] Application Management Department, JEOL Ltd, Tokyo, Japan.

[5] Faculty of Pure and Applied Sciences, University of Tsukuba, Ibaraki, Japan

*Corresponding author: Tamotsu Zako
E-mail: zako.tamotsu.us@ehime-u.ac.jp

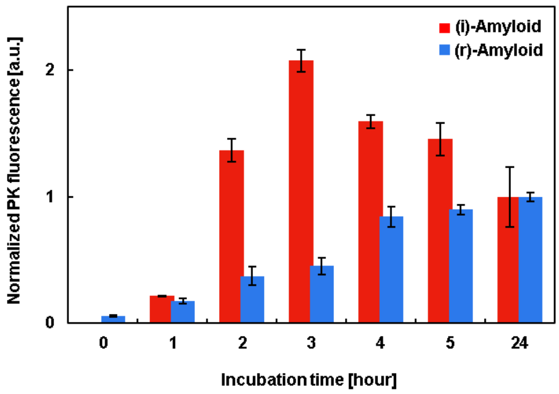

Figure S1 Fluorescence images (Fig. 1b) were analyzed by ImageJ. The fluorescence intensities of the spots (n=3) at the indicated time points were measured: (i)-amyloids (red) and (r)-amyloids (blue). The intensities were normalized for that of insulin amyloids incubated for 24 h.

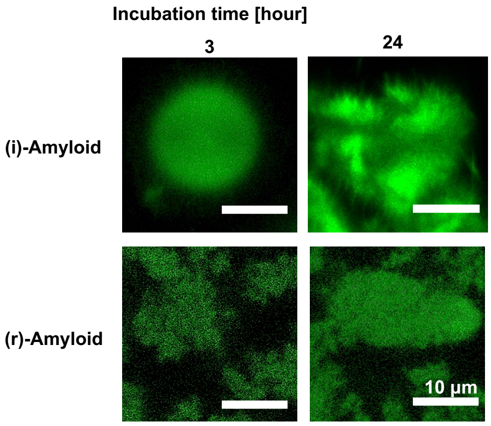

Figure S2 ThT fluorescence images of (i)-amyloids (upper) and (r)-amyloids (lower) at the indicated periods. The scale bar is 10 µm.

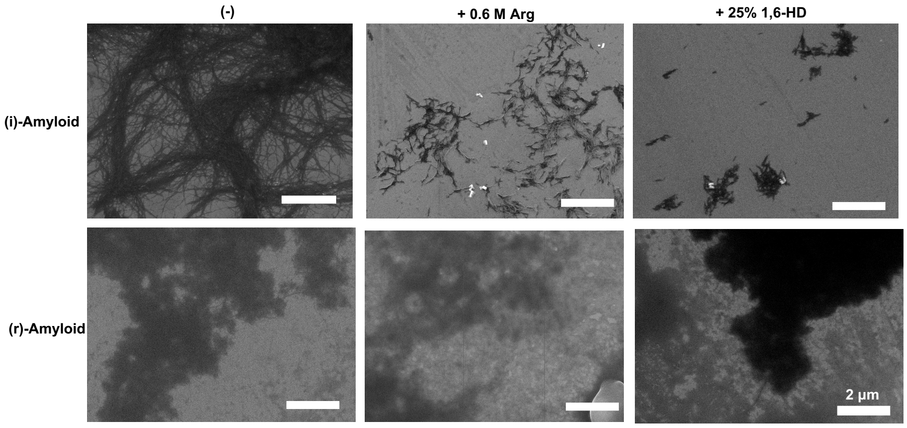

Figure S3 SEM images of (i)-amyloids (upper) and (r)-amyloids (lower) formed in the absence (left), the presence of 0.6 M Arg (middle), and 25 % 1,6-HD (right). The scale bar is 2 μm.

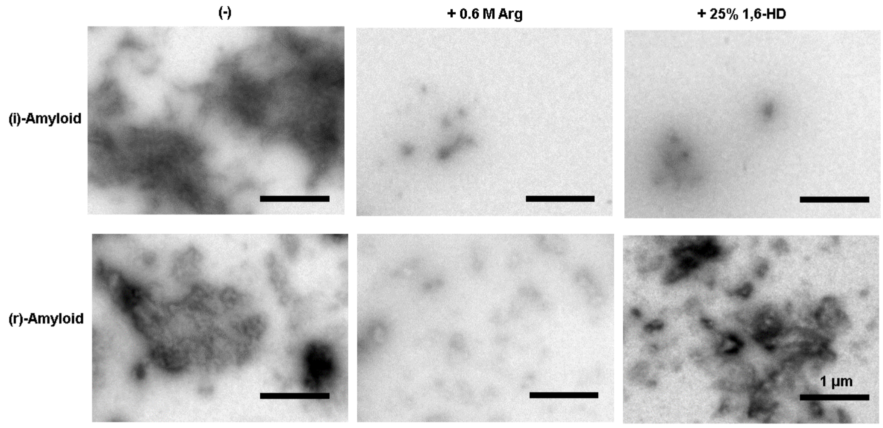

Figure S4 TEM images of (i)-amyloids (upper) and (r)-amyloids (lower) formed in the absence (left), the presence of 0.6 M Arg (middle), and 25 % 1,6-HD (right). The scale bar is 1 μm.

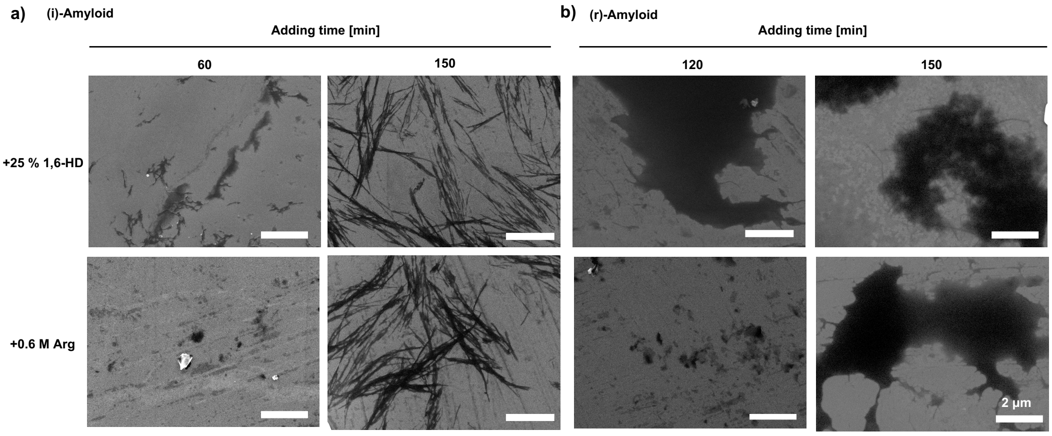

Figure S5 SEM images of (i)-amyloids **(a)** and (r)-amyloids **(b)** added with 25 % 1,6-HD (upper) or 0.6 M Arg (lower) at the indicated periods and incubated for total of 300 min. The scale bar is 2 μm.

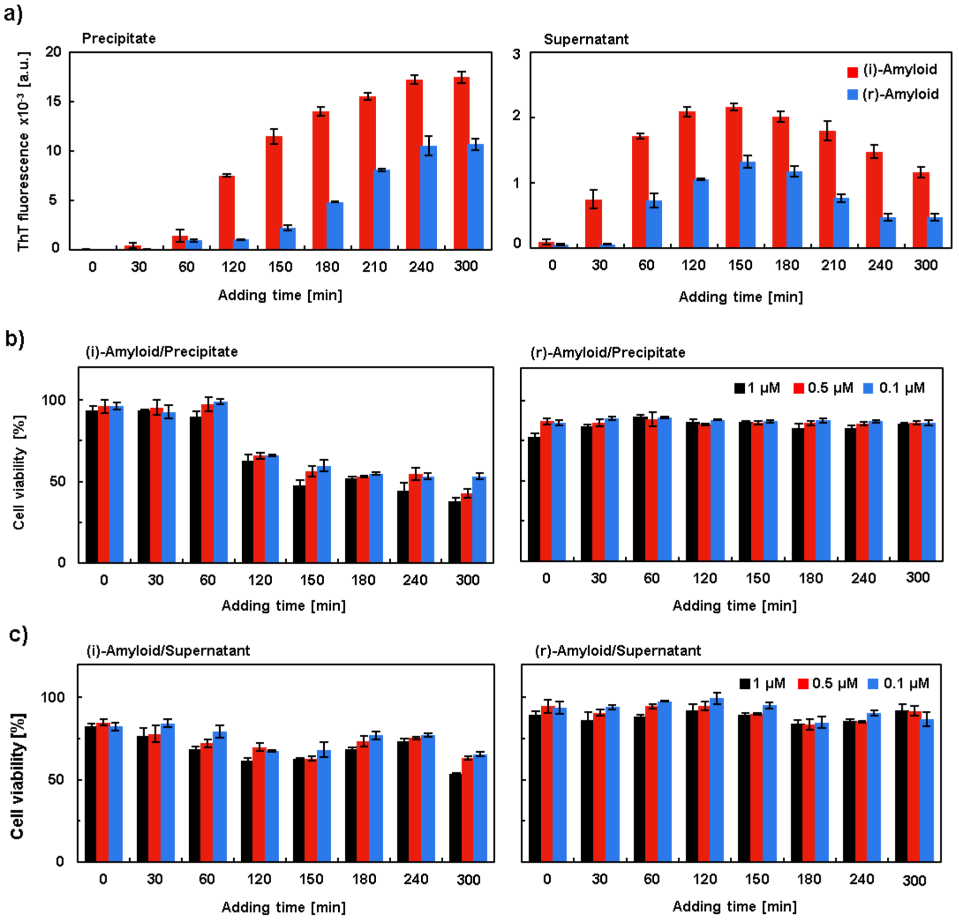

Figure S6 Evaluation of effect of Arg added on the formation process of the insulin amyloids
(a) ThT assay of the samples in the precipitate (left) and the supernatant (right) of (i)-amyloids (red) and (r)-amyloids (blue) with 0.6 M Arg added at the indicated time points. Peak fluorescence values at 490 nm (ThT) are shown. The protein concentration was adjusted to 5 µM.
(b, c) The cytotoxicity of the samples in the precipitate **(b)** and the supernatant **(c)** of (i)-amyloids (left) and (r)-amyloids (right) added with 0.6 M Arg at the indicated time points. The cytotoxicity was evaluated using the MTT assay against HeLa cells. Protein concentration: 1, 0.5, and 0.1 µM (black, red, and blue, respectively). The viability of cells with PBS addtion was normalized to 100%.

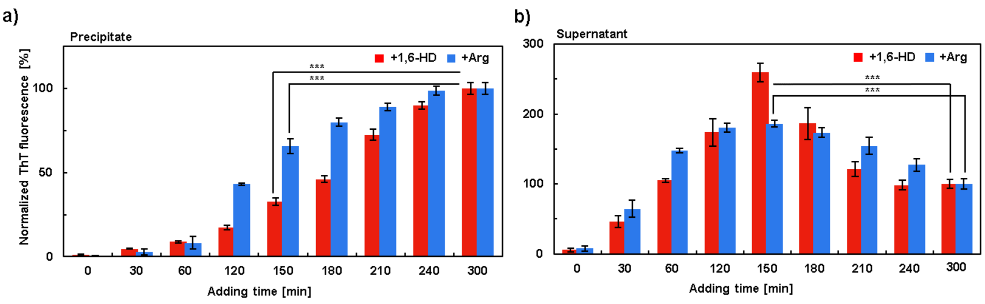

Figure S7 Effect of 1,6-HD and Arg on (i)-amyloid samples.
Normalized ThT intensity of the precipitate **(a)** and the supernatant **(b)** samples of (i)-amyloids with 25% 1,6-HD (red) or 0.6 M Arg (blue) added at the indicated time points and incubated for a total of 300 min was calculated from the data of Figure 4b and Figure S5. Protein concentrations were adjusted to 5 µM. The intensity in (i)-amyloids with the addition of 1,6-HD or Arg at 300 min was normalized to 100%. ***P<0.005 (two-tailed Student's t-test)

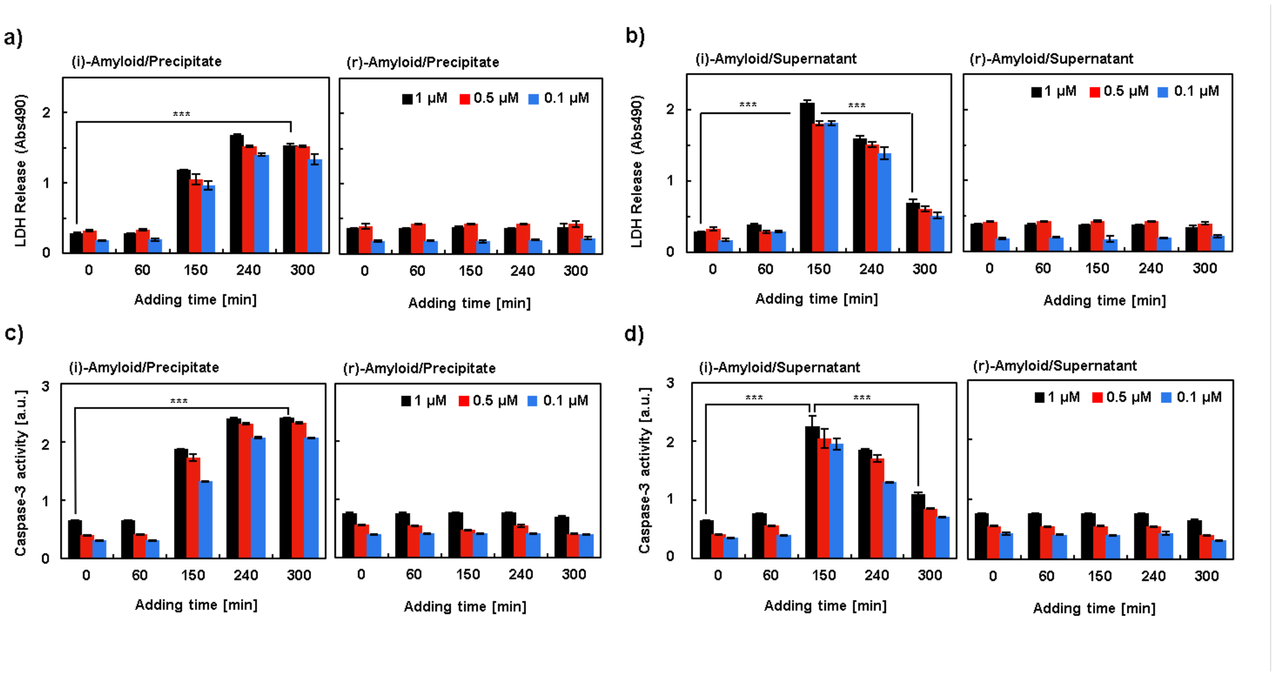

Figure S8 LDH assay **(a, b)** and Caspase-3 activity assay **(c, d)** of insulin amyloid samples added with 1,6-HD on the formation process.
The cytotoxicity of the samples in the precipitate **(a, c)** and the supernatant **(b, d)** of (i)-amyloids (left) and (r)-amyloids (right) with 25% 1,6-HD added at the indicated time points was evaluated using LDH assay **(a, b)** and Caspase-3 activity assay **(c, d)** against HeLa cells. The protein concentration: 1, 0.5, and 0.1 μM (black, red, and blue, respectively). ***P<0.005 (two-tailed Student's t-test)

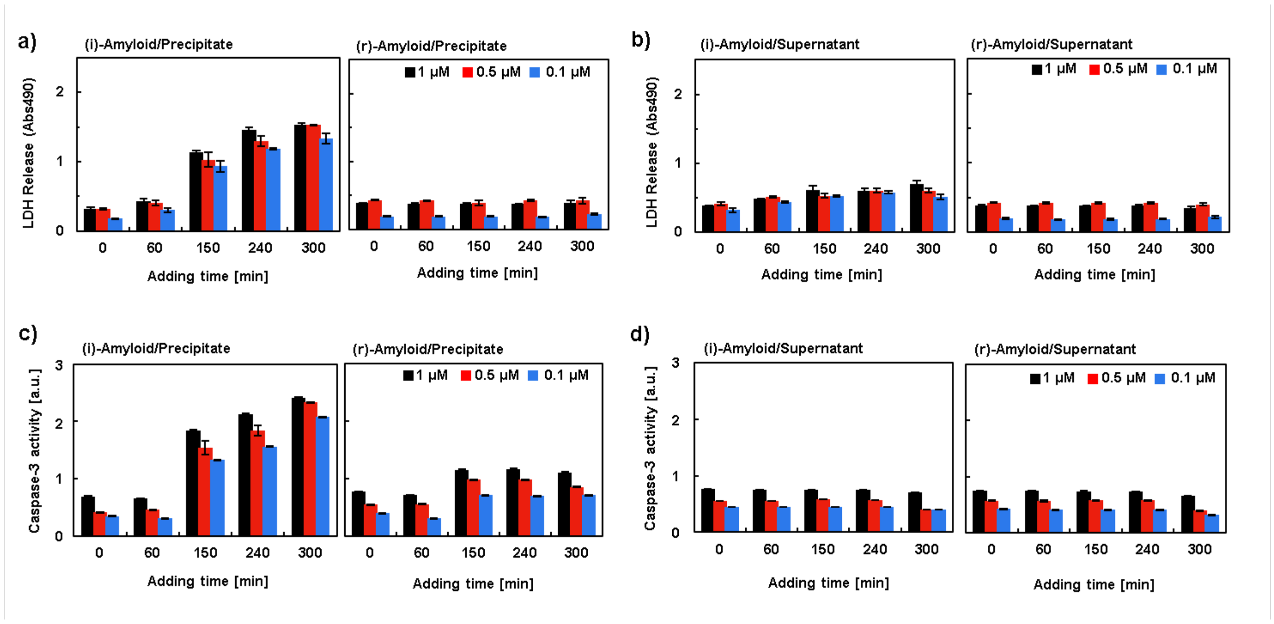

Figure S9 LDH assay **(a, b)** and Caspase-3 activity assay **(c, d)** of insulin amyloid samples added with Arg on the formation process.
The cytotoxicity of the samples in the precipitate **(a, c)** and the supernatant **(b, d)** of (i)-amyloids (left) and (r)-amyloids (right) added with 0.6 M Arg at the indicated time points was evaluated using LDH assay **(a, b)** and Caspase-3 activity assay **(c, d)** against HeLa cells. The protein concentration: 1, 0.5, and 0.1 μM (black, red, and blue, respectively).

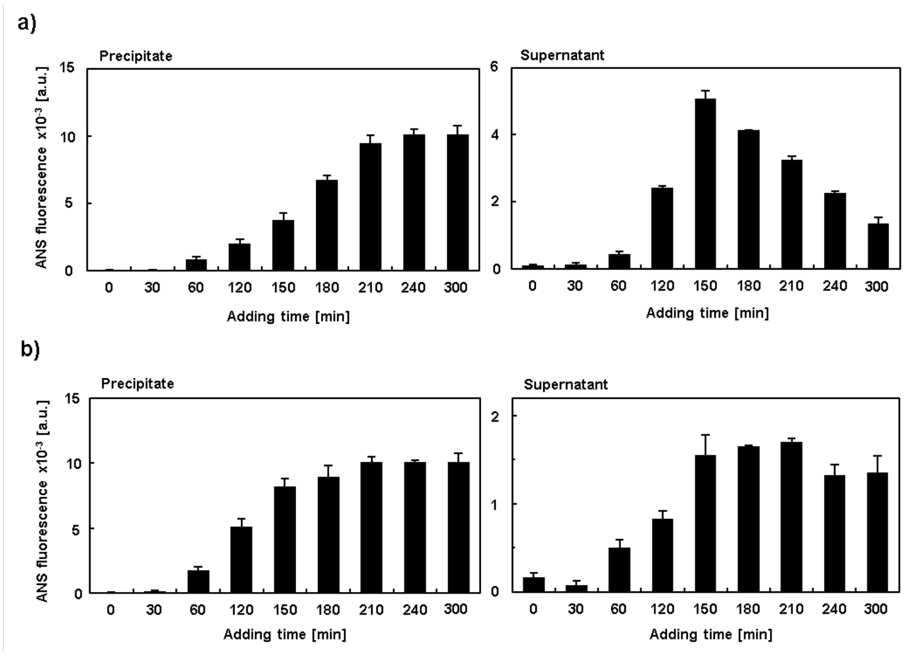

Figure S10 ANS assay of insulin amyloid samples added with 1,6-HD or Arg on the formation process. ANS fluorescence in the precipitate (left) and the supernatant (right) of (i)-amyloids added with 25% 1,6-HD **(a)** or 0.6 M Arg **(b)** at the indicated periods. Peak fluorescence values at 480 nm (ANS) are shown. The protein concentration was adjusted to 5 μM.

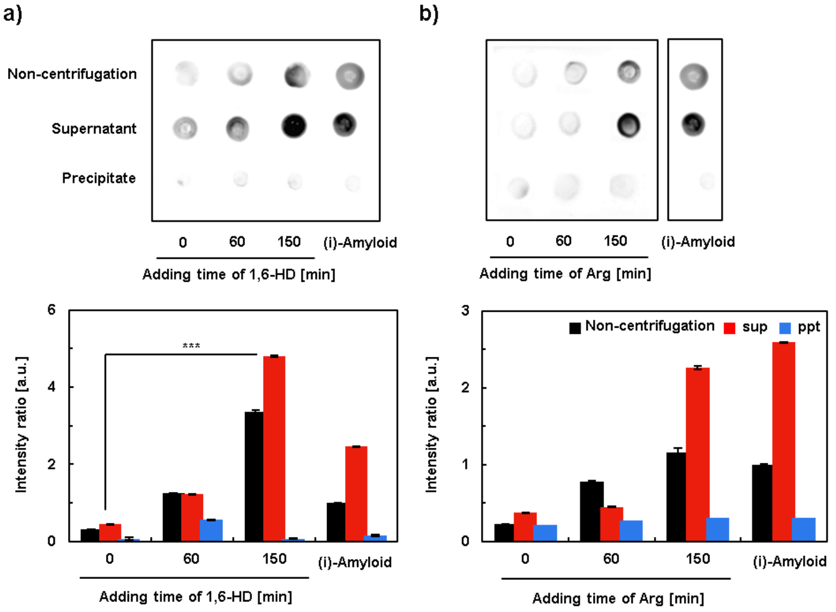

Figure S11 Dot blot assay of (i)-amyloid samples added with 25% 1,6-HD **(a)** or 0.6M Arg **(b)** at the indicated time points on the formation process by oligomer-specific antibody A11: the non-centrifuge (upper), the supernatant (middle), and the precipitate (lower) of (i)-amyloids. The (i)-amyloid control data in **(b)** was taken from **(a)**, which were stained in the same membrane. All samples were quantified by BCA assay and were diluted to the same protein concentration. The intensities obtained from the dot were shown as a graph: non-centrifuge (black), the supernatant (red), and precipitate (blue). Intensities were normalized to 100 % for non-centrifuge (i)-amyloid. The averaged intensities from the three different membranes were shown. ***P<0.005 (two-tailed Student's t-test)

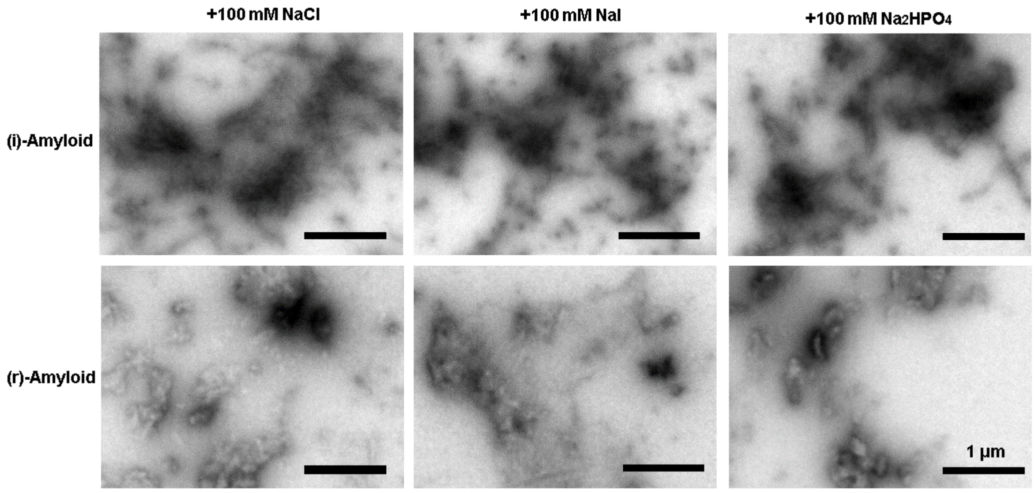

Figure S12 TEM images of the structure of (i)-amyloids (upper) and (r)-amyloids (lower) formed in the presence of 100 mM NaCl (left), NaI (middle), and Na_2HPO_4 (right). The scale bar is 1 µm.

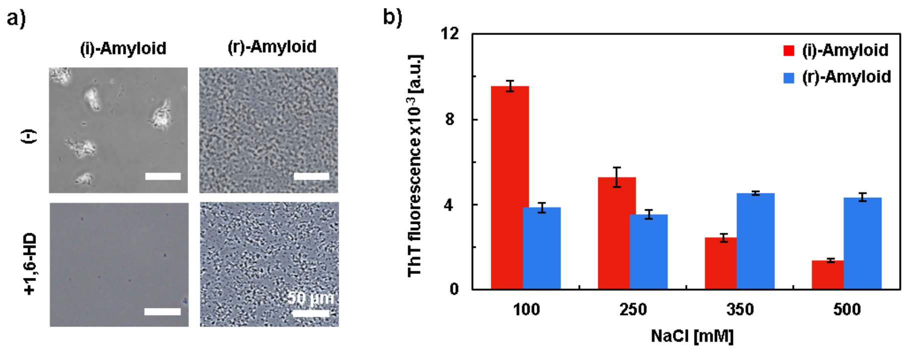

Figure S13 Formation of (i)-amyloids and (r)-amyloids at physiological conditions.

(a) Images of insulin amyloids were observed using an optical microscope. Insulin was incubated for 72 hours at 37°C and pH 7.4 with agitation (600 rpm) and was observed after the additives of 5 % 1,6-HD. The scale bar is 50 µm.

(b) ThT assay of insulin amyloids ((i)-amyloids (red) and (r)-amyloids (blue)) formed at 37°C and pH 7.4 with agitation (600 rpm) in the presence of NaCl. Peak ThT fluorescence values at 490 nm were shown. Protein concentrations were adjusted to 5 µM.

a)

original gel for Figure 2e

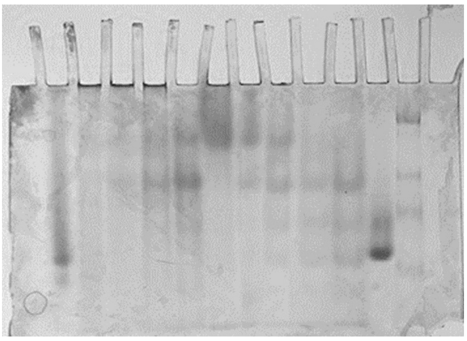

original gel for Figure 3f

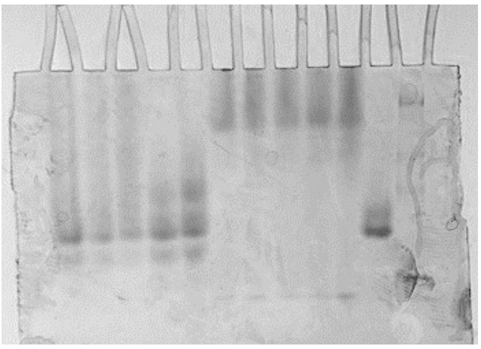

b)

original blot image for Figure S11a

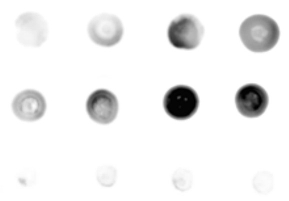

original blot image for Figure S11b

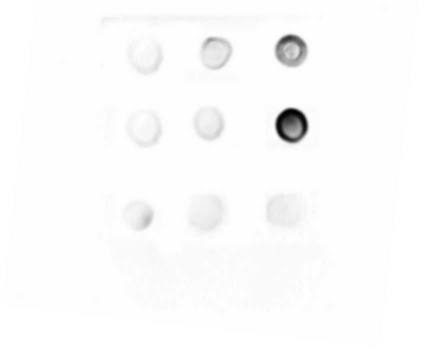

Figure S14 Original gel images (a) and blot images (b) used in the figure.
